# Multi-factorial modulation of colorectal carcinoma cells motility – partial coordination by the tetraspanin Co-029/tspan8

**DOI:** 10.18632/oncotarget.16247

**Published:** 2017-03-16

**Authors:** Yingying Zhu, Naouel Ailane, Monica Sala-Valdés, Farhad Haghighi-Rad, Martine Billard, Viet Nguyen, Raphael Saffroy, Antoinette Lemoine, Eric Rubinstein, Claude Boucheix, Céline Greco

**Affiliations:** ^1^ Inserm, UMR-S 935, SFR André Lwoff, Villejuif, France; ^2^ Université Paris-Sud 11, Paris, France; ^3^ Department of Oncology, Tongji Hospital, Huazhong University of Science and Technology, Wuhan, Hubei Province, China; ^4^ Inserm, UMS-33, SFR André Lwoff, Villejuif, France; ^5^ Inserm UMR-S 1193, SFR André Lwoff, Villejuif, France; ^6^ AP HP, Hôpital Paul-Brousse, Department of Biochemistry, Villejuif, France; ^7^ AP HP, Hôpital Necker, Department of Pain and Palliative Medicine, Paris, France

**Keywords:** Co-029/tspan8, colorectal carcinoma, cell motility, mycoplasmas, EGFR

## Abstract

Colorectal carcinoma cells Isreco1 display an ability to migrate controlled by a complex set of signals issued from the membrane. By comparing cells infected by mycoplasmas and mycoplasmas free cells, we have established that basal 2D migration is dependent on a double signal mediated by the collagen receptors integrins alpha1/2 and the Toll-Like receptor TLR2. The signal issued from mycoplasmas can be replaced by a TLR2 ligand and the functional effect is neutralized by silencing of MyD88. Following previous observation that downregulation of E-cadherin/p120 catenin increases cell motility, we now report that EGFR or CD44 inhibition have a similar effect on cell motility that is restricted to tetraspanin Co-029/tspan8 transduced IsrecoI cells (Is1-Co029). The modulation of cell migration linked to EGFR or CD44 can be neutralized by antagonizing Co-029 with the mAb Ts29.1 or by RNA interference. Altogether these data point to a crucial role of Co-029 in the modulation of colon cancer cell motility which could be related to the protumoral effect reported for this tetraspanin. Among surface molecules able to mediate Co-029 function, E-cadherin, EGFR and CD44 appear as likely candidates.

## INTRODUCTION

Cell motility has attracted considerable interest because of its involvement in normal tissue development and repair but also in tumor invasion and metastasis [1]. Frequently linked with proliferation and survival, this biological program has been defined as “invasive growth” [2, 3]. Depending on the cells and their environment, extracellular signals may trigger various aspects of this program. The obvious consequence of these biological activities is that they can be targeted for the treatment of tumors and are therefore widely investigated.

We have shown in a previous work that motility of the Isreco1 colon carcinoma cells is triggered by a collagen I substrate [4]. Whereas Isreco1 cells don't move on tissue culture plastic (TCP), they become intensely motile in an integrin dependent mechanism when a collagen I substrate is added. These cells display a regular pattern of migration as shown by single cell tracking that demonstrates a low dispersion of cell speed between individual cells. The expression of the tetraspanin Co-029/tspan8, that is considered to have a protumoral, prometastatic effect [5] does not change cell motility but induces an integrin switch and renders cells sensitive to the level of expression of the E-cadherin/p120ctn complex [4]. Indeed, Isreco1 basal cell migration relies on integrin α1β1 signaling for migration whereas Is1-Co029 cells are α2β1 dependent. Silencing of E-cadherin or p120ctn accelerates Is1-Co029 cells whereas no effect is found in Isreco1 cells. A physical association between Co-029 and E-cadherin has been demonstrated by crosslinking and the difference of response of the two cell lines has been shown to be RhoA dependent.

To find a possible molecular connection that would be a hint towards understanding the coordinating role played by Co-029 in cell motility, we used mass spectrometry (MS) for identification of the membrane proteins associated to the closely related tetraspanins Co-029 and CD9. The rationale for this approach is that tetraspanins build multimolecular complexes at cell surface [6–12] with a hierarchical organization in which each tetraspanin (mono- or homo/heteromultimeric) has one or more specific partner(s) to which it associates through protein-protein interactions (captured by chemical covalent cross-linking) and these primary complexes may associate together through tetraspanin-tetraspanin interactions involving membrane cholesterol and palmitoylation. These secondary interactions are revealed by immunoprecipitation of Brij97 lysates.

According to MS data, we have compared, in relation to the expression of Co-029, the contribution of EGFR, a major RTK signaling molecule, and CD44 that are both involved in tumor cell motility [1, 2], to determine how their modulation may influence this cellular function. Moreover, an unexpected observation made in the course of this work, i.e. the different pattern of migration of mycoplasma free versus mycoplasma contaminated cells, led us to analyze the role of TLR2 signaling.

## RESULTS

### Mycoplasms trigger migration of Isreco1 cells on collagen I

**Figure 1:**
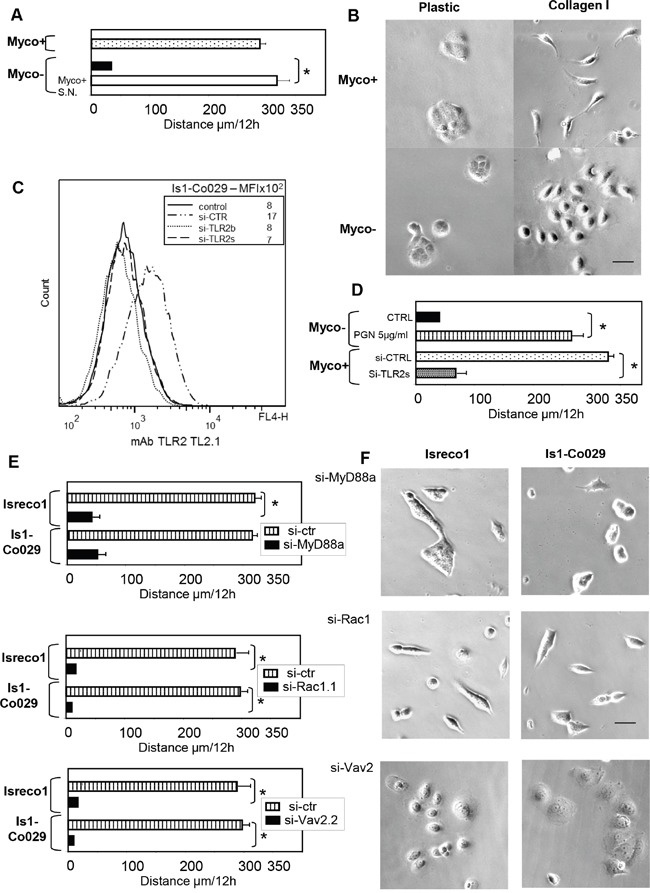
Mycoplasma infection triggers cell motility of Isreco1 cells on collagen I, involvement of TLR2 signalling **(A)** Opposite to Myco+ cells, mycoplasma free cells (Myco-) migrate slowly on collagen I substrate (black bar). Addition of mycoplasma infected supernatant (Myco+ S.N.) restores the migration of Myco- cells after 24 hours incubation (white bar). Averaged distance travelled by cells; error bars indicate s.e.m; asterisks indicates p≤.0001. **(B)** Cellular morphology according to mycoplasma status and culture substrate. **(C)** TLR2 expression and RNA interference. **(D)** The motility of Myco- (black bar) cells is restored by addition of the TLR2 ligand PGN (white bar) whereas RNA interference of TLR2 (si-TLR2s) inhibits migration of Myco+ cells (grey bar). **(E)** Silencing of Myd88, Vav2 and Rac1 strongly inhibit migration of Isreco1 and Is1-Co029 cells. **(F)** Silencing of MyD88, Vav2 and Rac1 induces different morphological aspects of Isreco cells that are all associated with profound inhibition of motility. For si-CTRL, a si-RNA directed to tetraspanin CD53 was used. Asterisks indicates p≤.0001.

Among multiple colon carcinoma cell lines tested for single cell migration ability on collagen I, only Isreco1 cells transduced or not with the tetraspanin Co-029 have a homogeneous pattern of cell migration with a low dispersion of cell speed between individual cells (Supplementary Movie 1 left). Isreco1 cells cultured on tissue culture plastic display a rounded morphology, don't move and build clusters of cells upon division whereas the same cells cultured on collagen I move rapidly with an elongated arc or fan shape [4]. Cell lines are regularly checked for the presence of mycoplasma and treated for one month with BM cyclin to remove mycoplasma infection. Upon treatment we observed usually that they nearly completely lost their ability of migration on collagen I (Figure 1A and Supplementary Movie 1). Moreover, mycoplasma free cells had a rounded and spread morphology when cultured on collagen very different from the fan shape morphology of mycoplasma contaminated cells (Figure 1B). The mycoplasma status of the Isreco cell lines was checked before and after treatment. Interestingly, if mycoplasma free cells were cultured with supernatant of infected cells, they recovered their migrating properties after 24 hours incubation and started to migrate at the same speed as untreated cells (Figure 1A). These observations indicate that migration of Isreco1 cells is triggered by two mechanisms, the first requiring an integrin-mediated signal triggered by collagen and a second signal that is mycoplasmas dependent. The expression of Co-029 does not modify the speed of the cells [4].

Since epithelial cells interact with microorganisms through pattern-recognition receptors as Toll-like receptors, we decided to explore this pathway. Among TLRs, TLR2 forming homodimers or heterodimers with TLR6 are the target of surface molecules of microorganisms such as lipopeptides and share the MyD88-mediated signaling pathway [13]. The presence of TLR2 in Isreco cells was demonstrated by immunofluorescence (Figure 1C), transcriptomal analysis and quantitative RT-PCR (data not shown). Moreover, we show that PGN, a TLR2 ligand, restores the motility of Isreco1 cells on collagen (Figure 1D and Supplementary Movie 2). Treatment of mycoplasma contaminated cells by TLR2 small interfering RNA (siRNA) led to 50% inhibition of cell migration on collagen (Figure 1D). To identify the mycoplasmas strains, we analyzed several immunoprecipitations performed in the following sections by mass spectrometry for contamination with mycoplasma proteins. We made a compilation of the 91 mycoplasma databases from Uniprot (Release-2016_05). Only proteins from mycoplasma hyorhinis could be detected with more than one specific peptide. The list of proteins is shown in Supplementary Table 1.

In order to identify downstream mediators of TLR2 signaling we investigated the NFkB pathway. We used as positive controls, the addition of TNFα or okadaic acid. Neither of these molecules induced a translocation of NFkB to the nucleus as judged by immunofluorescence using an anti p65NFkB antibody (not shown) or by immunoblot on subcellular protein fractions (Supplementary Figure 1). This suggests that the canonical NFkB pathway is defective in these cells. In agreement with this observation, we didn't observe cytoplasm to nuclear translocation in mycoplasma contaminated Isreco1 cells cultured either on plastic or collagen substrates or following stimulation by the TLR ligand PGN (Supplementary Figure 2). In contrast, silencing of MyD88 led to complete inhibition of cell migration (Figure 1E-1F and Supplementary Figure 3). Since MyD88 is required for cell migration in this model, we investigated a possible link between MyD88 and the small GTPase Rac1 which is essential for cell migration. Silencing of Rac1 led also to inhibition of cell migration (Figure 1E-1F and Supplementary Figure 3). In addition, Vav proteins have been reported to be critical mediators of LPS induced MyD88-dependent activation of Rac2, leading to the production of reactive oxygen intermediates [14]. The transcriptomal analysis of Isreco cells showed that only Vav2 is expressed (data not shown) and silencing of Vav2 in Isreco1 cells expressing or not Co-029 resulted in a complete block of cell migration in both cell types suggesting this protein as a link between MyD88 and Rac1 (Figure 1E-1F and Supplementary Figure 3).

As epithelial–mesenchymal transition (EMT) can promote tumor motility, we further investigated if the change of morphology and migration potential observed in cells cultured on collagen and either infected by mycoplasmas or stimulated by PGN was associated with molecular markers of EMT. For that purpose we first compared the expression of CK8/18 and vimentin by immunofluorescence in mycoplasma infected and mycoplasma negative cells. Despite the morphological changes when mycoplasma infected cells are plated on collagen, no disappearance of CK8/18 fibers was observed and the vimentin labeling was diffuse and punctuate but not filamentous whatever the cells, mycoplasma status or substrate plating (Supplementary Figure 4A). We also analyzed by quantitative RT-PCR four transcription factors associated with EMT. Twist and Zeb2 mRNA were not detectable in Isreco1(+/− Co-029) cells, whereas there was a generally moderate increase of Snail and Slug when cells were plated on collagen but independently of mycoplasma infection (Supplementary Figure 4B). This indicates that the mesenchymal aspect and behaviour resulting from the costimulation by TLR2 ligands and collagen I is not associated with a strong EMT at molecular level.

The SW480 cell line was chosen for *in vivo* experiments since they grow more rapidly and homogeneously than Isreco1 cells *in vivo*. Tumor size was similar in mice injected subcutaneously, independently of the mycoplasma status (Supplementary Figure 4C). At the histological level, no major differences were observed but tumors appear to be clearly delimited by a thin fibrous and cellular capsule that showed some signs of breaching in 2/5 mycoplasma infected tumors (data not shown). However, a possible interference of mycoplasma with tumor growth can't be completely ruled out since mycoplasma are progressively cleared *in vivo*. Indeed no mycoplasma could be detected in cultured cells obtained from three tumors issued from mycoplasma infected cells after at least 3 week development *in vivo*.

### Co-029 molecular partners

**Figure 2:**
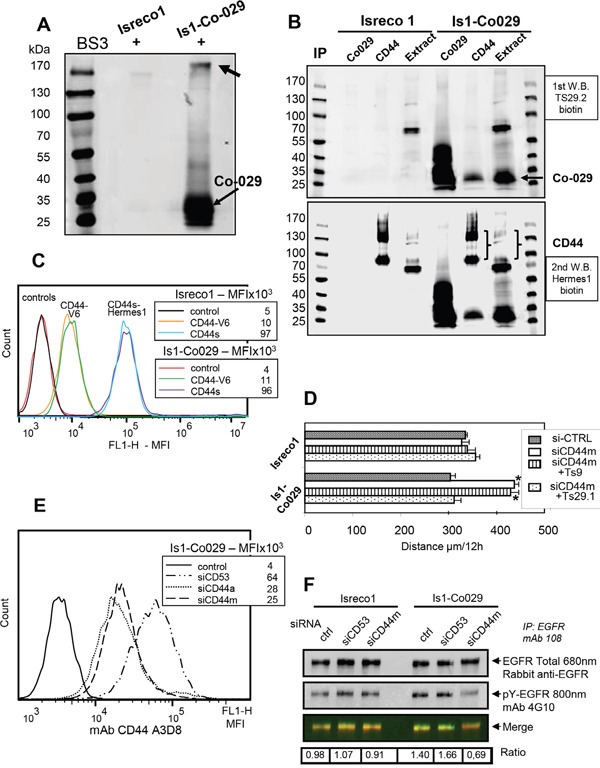
CD44 is associated with Co-029 **(A)** Crosslinking by BS3 of membrane proteins and immunoprecipitation (IP) by Ts29.1. Co-029 mAb shows by western blot the formation of a molecular complex above 170 kDa. **(B)** IP of Brij97 extracts shows co-precipitation of Co-029 by CD44 in Is1-Co029 cells (upper panel) whereas CD44 cannot be revealed in the Ts29.1 immunoprecipitation. **(C)** Expression of the CD44v6 isoform. **(D)** Increased motility induced by CD44 RNAi in Is1-Co029 cells that is reversed by Ts29.1 mAb and not CD9 mAb Ts9 (si-CD53 is used as si-CTRL). Asterisks indicates p≤.0001 (compared to control). **(E)** Efficiency of CD44 si-RNA. **(F)** A reduction of the ratio (arbitrary units) of pY-EGFR/total EGFR is observed upon CD44 RNAi treatment (ctr: no siRNA). EGFR was immunoprecipitated with mAb 108 and blotted with goat anti-EGFR and rabbit antigoat (680 nm) immunoglobulins followed by biotinylated 4G10 and streptavidin 800 nm.

In order to precise the molecular connection between Co-029 and membrane receptors involved in cell motility of mycoplasma infected cells, we have used MS to identify molecules that could be closely linked to Co-029 within the tetraspanin web [6–12]. Firstly, we tried to identify surface proteins that were crosslinked to Co-029 by membrane impermeant BS3. Since cross-linked Co-029 had been observed previously at an apparent MW of ∼200-220 kDa (Figure 2A) [4], we analyzed the content of the cross-linked product(s). We observed that one peptide belonging to the cdh3 domain of E-cadherin (382-GQVPENEANVVITTLK-398) and two peptides (682-LVINSGNGAVEDR-694, 716-ESSETPDQFMTADETR-731) from the cytoplasmic domain of CD44 were identified with a high score. This confirmed previous results suggesting an association between E-cadherin and Co-029 [4] and introduced CD44 as another possible Co-029 partner. The apparent MW of the crosslinked complex is higher than expected for a complex containing only E-cadherin or CD44s but it could be either a ternary complex with the 3 molecules or different complexes of Co-029 with homodimers of CD44 or E-cadherin. Subsequent MS analysis of the tetraspanins CD9 and Co-029 Brij97 complexes in Isreco1 and Isreco-Co029 cells showed the presence of 57 associated molecules, most of them already known to be present in the tetraspanin web (Table 1). Among these molecules, E-Cadherin (two peptides) and CD44 were found in both cell lines as demonstrated by their presence in cell extracts immunoprecipitated either by CD9 or by Co-029 antibodies. CD44, represented by 4 peptides (2 extracellular 79-YGFIEGHVVIPR-90 and 163-TNPEDIYPSNPTDDDVSSGSSSER-186 and the 2 peptides from the cytoplasmic domain found in crosslinking experiments), was found in the presence or absence of Co-029 in regions of the gels corresponding to the 80-90 kDa form considered as the CD44s (standard) form and to the 120-130 kDa form which is more likely representative of the epithelial isoform of CD44 (CD44E) containing alternative exons v9-v10 (Figure 2B). We also analyzed by MS CD44 immunoprecipitates in the presence or in the absence of Co-029. We detected previously mentioned peptides corresponding to CD44s but also peptides corresponding to alternative exons v8-v9 (498-TGPLSMTTQQSNSQSFSTSHEGLEEDKDHPTTSTLTSSNR-537) and v10 (546-DPNHSEGSTTLLEGYTSHYPHTK-568-V10) only in cells expressing Co-029 (data not shown).

**Table 1:** Fifty seven proteins found in the tetraspanin complexes of Isreco1 and Is1-Co029 cells after CD9 or Co-029 immunoprecipitation (IP)

Proteins (genes names)	Isreco1 IP CD9			Is1-Co029 IP CD9			Is1-Co029 IP Co-029		
	Peptides (n)		S-Area	Peptides (n)		S-Area	Peptides (n)		S-Area
	*Total*	*Unique*		*Total*	*Unique*		*Total*	*Unique*	
**Co-029/TSPAN8**	**NA**	**NA**	**NA**	**6**	**3**	**5,64E+09**	**6**	**4**	**1,10E+10**
CD9	11	6	1,20E+11	9	4	1,63E+11	10	4	9,39E+10
CD81	5	3	7,19E+09	5	3	1,79E+10	5	3	9,99E+09
ITGB1	29	18	2,72E+09	28	17	3,03E+09	27	16	2,43E+09
EPCAM	11	5	9,86E+08	12	7	2,62E+09	13	6	2,16E+09
ADAM10	24	17	1,78E+09	28	18	4,45E+09	24	16	2,15E+09
STX4	13	7	1,45E+09	7	2	2,78E+09	2	1	1,39E+09
PLP2	2	1	1,47E+09	1	1	2,28E+09	1	1	1,25E+09
SLC44A2	14	5	5,91E+08	17	8	4,44E+09	14	7	1,17E+09
**CD44**	**8**	**2**	**1,14E+09**	**9**	**5**	**5,61E+08**	**8**	**2**	**9,39E+08**
PTGFRN	38	18	1,52E+09	38	18	3,09E+09	30	15	4,52E+08
BCAM	20	15	4,54E+08	22	11	4,78E+08	20	14	4,35E+08
ITGA3	23	14	1,56E+09	23	13	1,89E+09	19	10	4,18E+08
GPR56	4	1	3,75E+07	2	1	1,93E+08	7	1	3,26E+08
CD82	4	3	9,79E+07	5	4	4,52E+08	5	4	2,96E+08
CYSTM1	2	1	1,59E+08	1	1	3,46E+07	1	1	2,61E+08
CD46	7	1	2,02E+08	5	1	4,65E+08	5	1	2,50E+08
LRRC59	10	6	2,45E+08	11	5	3,20E+08	10	3	2,23E+08
F3	7	5	8,17E+08	6	4	5,22E+08	5	2	2,22E+08
STX3	7	3	1,53E+08	8	3	1,93E+08	8	3	2,13E+08
GGT1	6	3	3,05E+08	5	2	1,79E+08	5	1	2,03E+08
CD63	1	1	1,36E+08	1	1	2,87E+08	4	1	1,61E+08
**EGFR**	**2**	**2**	**1,04E+07**	**21**	**8**	**1,41E+08**	**19**	**9**	**1,48E+08**
SLC3A2	10	3	3,68E+07	12	4	9,96E+07	18	7	1,48E+08
CDCP1	11	3	4,73E+08	19	8	5,11E+08	9	1	1,42E+08
TENM3	5	3	8,47E+07	3	1	7,78E+07	7	3	1,37E+08
EVA1A	3	2	5,56E+07	3	1	9,38E+07	2	1	1,35E+08
ST14	12	6	1,08E+08	11	6	2,10E+08	3	1	1,35E+08
ITGB4	39	21	4,28E+08	30	19	4,19E+08	35	18	1,25E+08
NT5E	10	4	5,20E+07	12	6	6,67E+07	15	5	1,25E+08
GPR110	4	1	2,30E+07	2	1	6,26E+07	3	1	9,68E+07
GPC1	2	1	1,96E+07	4	1	5,88E+07	6	3	8,96E+07
CD97	7	3	5,86E+07	5	1	5,70E+07	7	6	8,75E+07
TSPAN5	3	1	5,47E+07	1	1	1,62E+08	1	1	8,57E+07
ATP1B1	11	3	2,98E+08	6	2	1,76E+08	4	1	8,51E+07
ITFG3	1	1	2,62E+06	5	3	3,21E+07	7	4	8,17E+07
BSG	4	4	2,32E+07	6	1	7,72E+07	3	1	8,15E+07
TSPAN14	5	2	2,79E+08	10	4	7,02E+08	4	2	7,97E+07
ITGA6	33	15	4,77E+08	41	21	3,50E+08	29	12	7,62E+07
GPR126	2	2	6,91E+06	7	3	7,35E+07	6	1	7,61E+07
STX7	5	3	9,34E+07	4	2	8,99E+07	5	1	7,14E+07
IGSF8	7	1	5,46E+08	13	5	3,29E+08	4	1	5,83E+07
ADAM17	6	1	2,75E+07	10	3	2,81E+08	6	2	5,24E+07
PCDH1	6	3	1,47E+07	6	1	2,88E+07	11	3	4,70E+07
STX2	9	6	2,93E+07	6	4	4,90E+07	8	4	4,64E+07
LMAN2	3	2	3,04E+06	6	1	4,09E+07	7	3	3,97E+07
SDC4	2	1	7,79E+06	1	1	1,18E+07	2	1	2,46E+07
ATP1B3	4	3	8,39E+07	5	3	4,55E+07	2	1	2,43E+07
SLC44A1	9	7	5,25E+07	10	5	2,04E+08	2	1	2,26E+07
PLAUR	3	1	3,22E+07	4	1	3,84E+07	3	2	1,68E+07
HLAcII	2	3	1,33E+07	1	4	2,51E+07	3	4	1,61E+07
CLDN1	2	1	1,91E+07	2	1	1,50E+07	2	1	1,46E+07
PLSCR3	1	1	1,51E+07	1	1	1,16E+07	3	1	1,42E+07
L1CAM	18	6	5,79E+07	5	1	6,11E+06	10	3	1,17E+07
LDLR	5	4	5,08E+06	4	2	1,32E+07	4	2	1,09E+07
**CDH1**	**4**	**2**	**2,25E+07**	**3**	**1**	**1,38E+07**	**2**	**1**	**8,10E+06**
MCAM	12	7	3,38E+07	3	1	8,78E+06	1	1	5,69E+06
NOTCH2	5	1	7,98E+06	3	1	1,30E+07	5	3	5,07E+06

MS identification of membrane proteins (GPI anchor and single or multipass membrane proteins) associated to CD9 and Co-029 after IP by Ts9 (CD9) or TS29.2 (Co-029) mAbs of Brij97 extracts from Isreco 1 and Is1-Co029 cells cultured on collagen. Proteins were ordered by decreasing label free quantitation of identified proteins in Is1-Co029 Ts29.2 IP. Co-029 found only in Is1-Co09 cells was added for information. Proteins studied in this article are indicated in bold.

EGFR and several integrins were also found in CD9 and Co-029 immunoprecipitates but not the collagen receptors integrins α1β1 and α1β2 (a weak association of α1β2 was found in the CD9 immunoprecipitate). Additional MS analysis of cells cultured on tissue culture plastic were performed to determine if collagen I changed the characteristics of integrins/tetraspanins complexes obtained from cells cultured on this substrate. The label free quantification (S-Area) shows that concerning the major associated integrins (ITGA3, A6, AV, ITGB1, B4), the association is in the same order of magnitude between cells cultured on tissue culture plastic vs collagen I but unexpectedly, for ITGA3, A6 and B4, the number of identified peptides is much higher in the CD9 co-immunoprecipitations when cells are seeded on collagen I (Supplementary Table 2). For EGFR eight and nine unique peptides were identified in co-precipitates from Is1-Co029 cells by mAbs Ts9 (CD9) and Ts29.2 (Co-029) respectively and only 2 peptides by Ts9 in coprecipitates from Isreco1 cells. Label-free quantification varied accordingly suggesting a greater association of EGFR with the tetraspanin enriched microdomains in Is1-Co029 cells Table 1.

### Role of EGFR on cell migration

**Figure 3:**
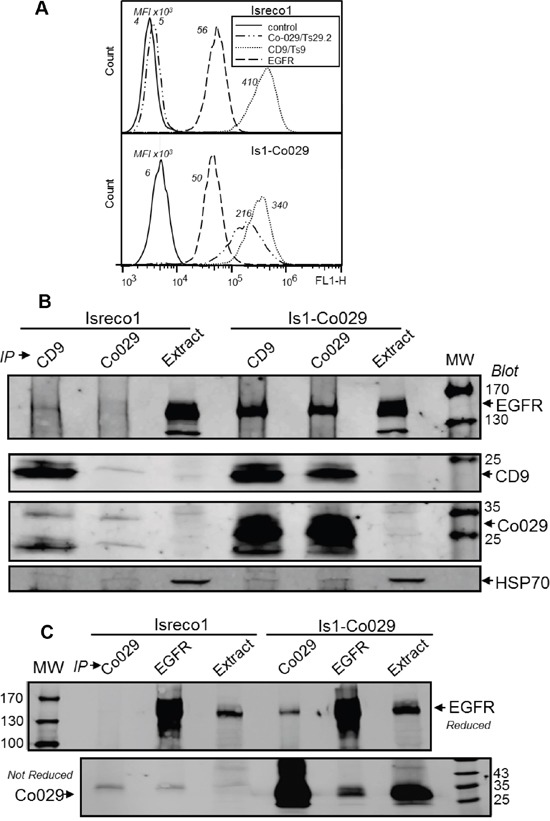
Co-029 increases the association of EGFR with the tetraspanin web **(A)** Co-029, CD9 and EGFR flow cytometry profiles of Isreco1 cells and Co-029 transduced cell line Is1-Co029. Mean fluorescence intensity (M.F.I.) values are indicated. Background represents the signal detected with a negative control mAb. The level of EGFR is similar in the two cell lines. **(B)** The presence of Co-029 increases the association of EGFR with the tetraspanin web as shown by the clear co-precipitation of EGFR with CD9 and Co-029 in Is1-Co029 cells whereas only a weak co-precipitation with CD9 is seen in Isreco1 cells. **(C)** Immunoprecipitation of EGFR by mAb 108 shows the reciprocal association of Co-029 with EGFR.

**Figure 4:**
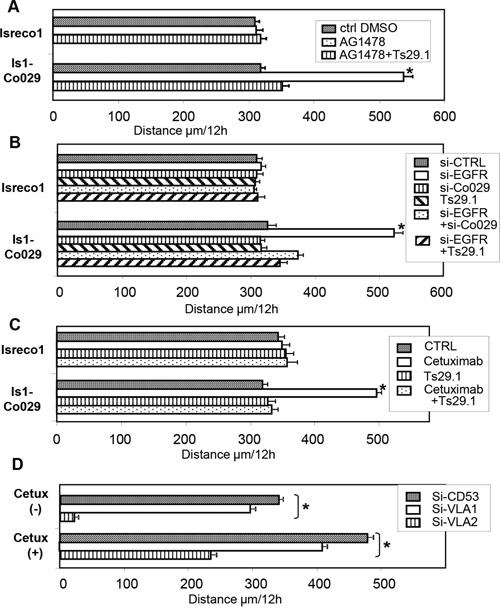
The effect of EGFR on cell motility is related to Co-029 expression **(A)** Increased motility of Is1-Co029 cells induced by chemical inhibitor of EGFR, AG1478, **(B)** by silencing of EGFR, and reversion by Co-029 silencing or addition of the Co-029 mAb Ts29.1 (for si-CTRL = siCD53). **(C)** Increased motility induced by Cetuximab and reversion by addition of the Co-029 mAb Ts29.1. **(D)** Inhibition of Is1-Co029 cell motility by integrins VLA1 and VLA2 RNAi. Asterisks indicates p≤.0001.

**Figure 5:**
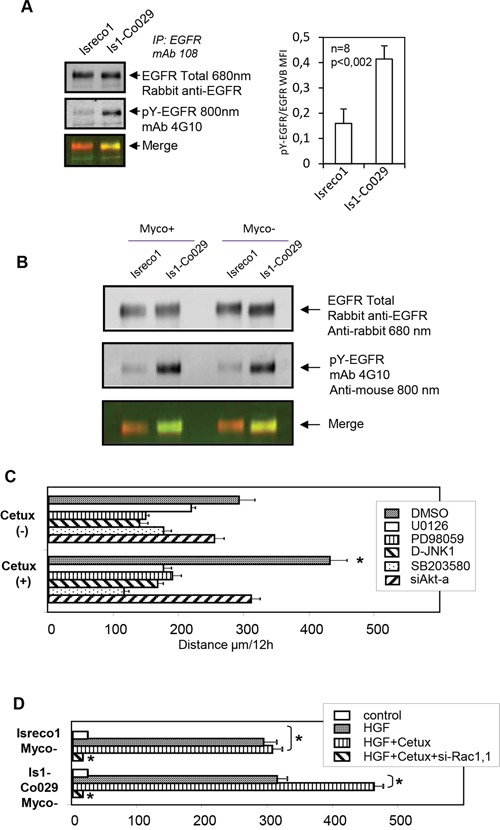
EGFR phosphorylation and signalling pathways **(A)** Basal tyrosine phosphorylation of EGFR is increased in Is1-Co029 cells as shown by repeated (n=8) immunoprecipitations of EGFR and labelling of pY by mAb 4G10. **(B)** Increased phosphorylation of EGFR upon Co-029 expression is independent of mycoplasma status. **(C)** The chemical MAPK inhibitors and Akt RNAi reversed the acceleration of Is1-Co029 cells induced by Cetuximab. **(D)** HGF triggers cell migration of Myco- cells on collagen I. Effect of Cetuximab and Rac1 RNAi (si-Rac1.1). All tests (except for si-Rac1.1) were performed with control si-RNA (si-CD53). Asterisks indicates p≤0001.

EGFR is detected on Isreco cells by membrane immunofluorescence (Figure 3A) and in agreement with MS data, we confirmed the association of EGFR with CD9 and Co-029 by western blot performed after cell lysis with Brij97 detergent (Figure 3B). However, in the presence of Co-029 the association of EGFR with the tetraspanin microdomains is increased 8 to 15 fold as shown by quantification in MS (label free) and in immunoblot. Reciprocally, Co-029 is coprecipitated with EGFR from Is-Co029 cells (Figure 3C). EGFR kinase inhibitor AG-1478 (Figure 4A) or EGFR silencing (Figure 4B) or Cetuximab (a humanized monoclonal antibody that blocks EGFR) (Figure 4C), all induced an increase of cell motility of Is1-Co029 cells without a detectable effect on Isreco1 cells. The motility enhancement associated with EGFR inhibition was reversed either by Co-029 silencing or by co-treatment with the Co-029 mAb Ts29.1. The increased motility of Is1-Co029 cells (induced by Cetuximab), as well as basal cell motility on collagen, are strongly inhibited by VLA2 RNA interference (RNAi), whereas VLA1 RNAi has only a moderate effect (Figure 4D). To explore the differential effect of EGFR inhibition on cell motility, we analysed basal EGFR phosphorylation with the anti-pY mAb 4G10. It appears that phosphorylation of EGFR in Is1-Co029 cells is higher than in Isreco 1 cells (Figure 5A), independently of the mycoplasma status (Figure 5B), suggesting that the activation of EGFR may have an inhibitory effect on cell migration that is compensated by other mechanisms since both cell lines migrate at approximately the same speed. Since these cells have a KRAS mutation, it is supposed that the EGFR effects on cell migration are Erk independent. However, as in our previous work [4], we show a limited but significant inhibitory effect of ERK, p38 and JNK inhibitors on cell motility in cells expressing Co-029. Inhibitors of the MAPKinases pathways also impaired the stimulatory effect of Cetuximab and reduced the migration of Cetuximab treated cells below basal level values (Figure 5C). A similar but less pronounced effect was obtained by Akt inhibition (Figure 5C). Interestingly, it was possible to substitute the effect of mycoplasma on cell motility by HGF addition (Figure 5D), whereas EGF had no effect (not shown). As for mycoplasma infected cells, Cetuximab increased only the motility of Co-029 expressing cells. Cells were completely stopped by Rac1 RNAi.

### Role of CD44 on cell migration

Western blots of cell extracts or immunoprecipitates of CD44 showed 2 major isoforms at 80-90 and 130 kDa and other forms with higher apparent MW (Figure 2B). We confirmed CD44/Co-029 association by immunoblot performed after cell lysis with Brij97 detergent and co-immunoprecipitation. Since Co-029 association was observed by immunoblot in the CD44 immunoprecipitation but not in the reverse precipitation, it precluded the observation of a preferential association of Co-029 with a specific isoform of CD44 (Figure 2B) such as CD44v6. CD44v6 was expressed at the same level in Isreco cell lines independently of the expression of Co-029 (Figure 2C).

The association between Co-029 and CD44 may be functionally relevant since CD44 RNAi (Figure 2D-2E) induced an increased motility of Is1-Co029 cells, reversed by the anti Co-029 mAb Ts29.1, whereas no effect of CD44 RNAi was found in the Isreco1 cell line (Figure 2D). We investigated whether CD44 effect could be mediated through an interaction with EGFR by measuring EGFR phosphorylation in CD44 siRNA treated cells. Reduction of CD44 expression was accompanied by a >50% reduction of EGFR phosphorylation specifically in Is1-Co029 cells (Figure 2F).

## DISCUSSION

In this work we demonstrate that the migration on collagen I of colorectal carcinoma cells Isreco1 and its Co-029 transduced counterpart, Is1-Co029, is a complex and multistep event with differences linked to the expression of Co-029. In both types of cell lines, a double signal is required for induction of motility analyzed at single cell level. Collagen I triggers a signal mediated by β1 integrins involving the collagen receptors α1 and α2 [4], whereas a 2^nd^ signal mediated by MyD88 is necessary. This signal appears to be triggered by mycoplasma in contaminated cells and can be substituted by PGN, a TLR2 ligand, in mycoplasma free cells. A second level of control over cell motility is exerted by Co-029 through different membrane receptors. In a previous work, we already showed that when Co-029 is expressed, the speed is controlled by the E-cadherin/p120ctn complex since silencing of these proteins increases the speed of the cells. In this work we also show that when Co-029 is expressed, EGFR or CD44 inhibition increases cell motility.

If the involvement of viruses and bacteria has been widely recognized as key event for the development of several types of tumors, the role of mycoplasma has been more rarely addressed. This class of bacteria that lacks a cell wall has the particularity to grow in close relationship with mammalian cells apparently for unlimited time and without major changes in cell aspect. For biologists, mycoplasmas are generally considered as a drawback in experiments dealing with cell cultures since they can interfere with multiple parameters that may influence results of experimental investigations. For that reason, it is generally requested to work with mycoplasma free cells and several types of antibiotics are able to clear the cells with varying efficiency. However, the effect of mycoplasmas derived molecules on migration and invasion of normal or malignant cells has already been suggested by some experimental data. Several studies have reported that mycoplasma infection could increase tumor cell invasiveness [15], and the presence of Mycoplasma hyorhinis protein p37 and Mycoplasma DNA in more than 50% of human cancer tissues including ovarian, gastric, colon carcinomas, esophageal, lung or breast cancer vs less than 25% of benign digestive lesions indicates the frequent coexistence of mycoplasma and tumor *in vivo* [16]. Mycoplasma may enhance the malignant phenotype in prostate and melanoma cancer cells through increased invasion and migration potential [17]. A role for the mycoplasma protein p37 has been shown by neutralization with the corresponding antibody [17]. Even a direct role of mycoplasma in cell transformation has been inferred from cultures of benign human prostate cells (BPH1) infected with M. genitalium or M. hyorhinis that led to the acquisition of a malignant phenotype with anchorage independent growth, increased migration and invasion [18]. These phenotypic alterations were associated with the acquisition of chromosomal aberrations. A statistically significant association between M. hominis infection and human prostate cancer has been reported [19–21]. In the neuroblastoma cells SH-SY5Y, an upregulation of calpastatin was reported to result in the inhibition of calpain, a proteolytic enzyme involved in many biological processes including migration [22]. Accordingly, the phenotypic alterations induced by mycoplasmas could be viewed as tumor promoting events or even as tumorigenic.

Results in our model demonstrate that mycoplasma have a direct triggering effect on cell migration and that this effect is mediated by TLR2. Physiologically, epithelial cells are continuously exposed to pathogens for which they have developed defense mechanisms in order to maintain the integrity of the tissue barrier [23]. They express different pattern recognition receptors as TLR or Nod that upon ligand binding and corresponding signalization lead to the production of host defense molecules. In addition TLR2 and 5 signaling may induce epithelial repair and survival independently of inflammatory cells or mediators [24]. Through their ability to activate the adaptive immune system, TLR may act as negative regulators of tumors. On the other hand, a direct effect of TLR ligands may promote tumor survival and growth *in vitro* and *in vivo* (for review see [25]). For instance the endogenous ligand versican may activate the TLR2 pathway [26]. Whether the ability of mycoplasmas to trigger cell migration *in vitro*, observed in the present report, could be viewed as rendering tumor cells more invasive and aggressive in *in vivo* situations is not supported by tumor growth in subcutaneously injected nude mice. However the absence of differential growth after subcutaneous implantation of mycoplasma infected or mycoplasma free cells may be explained by the observation that infected cells are cleared from mycoplasma *in vivo*.

Another level of regulation in the mechanisms of migration of Isreco1 cells is added by the stimulatory effects of inhibitors of EGFR only in cells expressing the tetraspanin Co-029. In this multiparameter control of motility, it is interesting to observe that the tetraspanin Co-029 seems to connect EGFR with the signaling pathways involved in the dual Integrin/TLR induced cell motility since silencing of EGFR accelerates migration of Co-029/tspan8 expressing cells.

In an effort to find a link between modulation of cell motility and Co-029 expression we undertook an extensive MS analysis of Co-029 associated molecules. We compared data obtained from chemical crosslinking experiments that identify primary partners with those issued from immunoprecipitation of Brij97 extracts that harvest higher order complexes (tetraspanin web). In addition to E-cadherin, crosslinking suggests that Co-029 associates with CD44 but in a non-exclusive way since CD44 is found in the tetraspanin web (Brij97 extracts) also in the absence of Co-029. CD44 is a very complex surface molecule due to its multiple spliced isoforms and heavy glycosylation. Its extra- and intracellular domains are known to interfere with numerous molecules particularly after fixation of hyaluronate. CD44 is associated with major cell functions like adhesion, motility, proliferation, stemness and is, as such, an actor of the tumor phenotype [27]. Among CD44 functionally interacting molecules, RTK, matrix proteinases and ERM are noticeable and could represent a link between cell motility and Co-029 expression.

Concerning the association of Co-029 with EGFR, MS analysis and western blots of Brij97 extracts immunoprecipitated with Ts9 (CD9) and Ts29.2 (Co-029) clearly show an increased association of EGFR with the tetraspanin web in the presence of Co-029. To our knowledge, it is the first time that the association of EGFR to the tetraspanin web was shown using the detergent Brij97 that is much more stringent than Brij98 or Chaps used in previous studies [28–30]. However, since EGFR was not crosslinked to Co-029 by BS3, a direct interaction is not proven.

The increased motility induced by the inhibitor AG-1478 and by siRNA, directed against EGFR, is reproduced by a function blocking antibody, suggesting that EGFR's signal in Co-029 expressing cells may occur through an autocrine stimulation pathway. Indeed, increased phosphorylation of EGFR in Is1-Co029 cells compared to Isreco1 cells indicates an activation (or transactivation) of EGFR. The case of EGFR is particular since the mutation of KRAS should result in constitutive stimulation of the Erk1/2 pathway, rendering these cells resistant to EGFR issued signals. Our results show an unanticipated effect of silencing or functional blocking of EGFR that activates cell migration, indicating that in this configuration (constitutive activation of the Erk1/2 pathway), with the cooperation of Co-029, EGFR signaling may act as a brake of cell migration, possibly by Erk1/2 induced negative feedbacks [31].

The control of RTK signaling by the tetraspanin web has been reviewed extensively recently [32, 33]. Depending on the cell type and the tetraspanin tested, different effects may be observed, for instance CD151 enhances ErbB2 signaling [34], whereas CD82 attenuates EGFR signaling [28, 35, 36]. CD82 expression in several cell lines has been shown to modify signaling events downstream of integrins and EGFR [28, 35, 36]. A functional interaction between CD9 and α2β1 integrin has also been reported [37].

More generally, crosstalk between integrins and RTK signaling pathways has been largely documented in the literature [38]. This crosstalk may vary between different cell types but in certain cells, a significant fraction of integrin signaling may be mediated by RTK transactivation. Furthermore, the EGF induced phosphorylation of EGFR is potentiated by integrin-mediated adhesion but diminished by the integrin interacting tetraspanin CD151 [28, 36, 39].

On the whole, crosstalk involving tetraspanins may start at the membrane level through interactions within the tetraspanin web but also downstream, along signaling pathways that are differentially connected/activated according to membrane organization and signals issued from the microenvironment.

In Is1-Co029 cells, we have shown that the expression of Co-029 is dramatically involved for regulation of 2D cell migration through E-cadherin/p120catenin [4], EGFR or CD44. On the contrary, in Isreco1 cells, these molecules do not seem to be involved in the regulation of basal cell migration, at least within the limits of this methodology. An explanation could be proposed in view of the hierarchical organization of the tetraspanins web in which the presence of Co-029 would preferentially interconnects adhesion receptors and RTK signaling pathways to drivers of cell migration such as TLR2 and integrins, possibly through the multifaceted molecule CD44. This interpretation remains compatible with the nanocluster organization of tetraspanin/partners complexes visualized by super-resolution microscopy [40] that challenges the view of the microdomains based organization of the tetraspanin web. If in this example Co-029 serves as an integrator of signals regulating cell migration, it may explain why and how different cell types may respond differently to similar stimuli since this tetraspanin has a restricted tissue expression and its expression varies between tumors of same tissue origin. A limit to the interpretation of the data is the 2D *in vitro* model of cell migration used in this work that may not be representative of *in vivo* tumor cell migration in which 3D migration and multiple molecular substrates introduce different parameters. Finally, since biological effects linked to the modulation of adherence molecules or RTK may be reversed by Co-029 antibodies, this points to possible mechanisms of the *in vivo* efficiency of these antibodies in experimental models [41, 42].

## MATERIALS AND METHODS

### Cell lines

The cell line Isrecol was initially derived from a primary human colon cancer (Duke's C, class III) surgical specimen [43]. These cells, transferred to our laboratory in 2002 by Dr. B. Sordat at ISREC, Lausanne, were cultured in Dulbecco's modified Eagle's medium (DMEM) supplemented with 10% FCS, glutamax and antibiotics (all from Invitrogen). For experiments, tissue culture plastics were coated with rat tail collagen I (BD Biosciences) for 1 hour at 50μg/ml and rinsed with PBS. Isreco1 cells were transduced to express the tetraspanin Co-029. Non cloned cell lines were used for these experiments and are called Is1-Co029 [4]. Phenotypic characteristics of the Isreco cell lines (morphology and surface markers) were reported previously [10]; they are checked after each thawing and before a set of experiments to avoid contamination between cell lines. To determine the mutational status of the cells we used OncoCarta^TM^ Panel v1.0 from Sequenom with the MassARRAY system. The Isreco1 and Co-029 transduced cells harboured a G12D homozygous mutation of KRAS whereas no mutations of BRAF and PI3K were found. The cell lines were further characterized by transcriptomal analysis (Miltenyi Biotech Microarray Service, Bergisch Gladbach, Germany) using Agilent Whole Genome Oligo Microarrays (4×44K one color). For *in vivo* studies, we used the SW480 cells (ATCC) that were checked for the homozygous G12V mutation before use.

### Mycoplasma detection

Mycoplasma infection was searched by using the Mycoplasma Detection Kit MycoAlert® (Lonza) which detects the presence of ATP in the supernatant.

### Antibodies

The anti-tetraspanin mAb Ts9 against CD9, Ts29.1(IgG1) and Ts29.2(IgG2b) directed against Co-029 used in this study were produced in our laboratory [4]. The following commercial antibodies were purchased: anti p65-NFkB (Goat IgG and mouse monoclonal, Santa-Cruz), anti EGFR (1005) (Rabbit IgG, Santa-Cruz), anti EGFR Cetuximab (Merck Serono), anti EGFR mAb108 (ATCC), monoclonal biotinylated anti CD44 156-3C11 (Thermo Scientific), monoclonal anti CD44 Hermes-1 (rat, Thermo Scientific), monoclonal anti CD44v6 VVF8 (Mouse, Abcam), anti-Cytokeratin 8 and 18, anti-Vimentin (Invitrogen, France). These antibodies were used as primary antibodies for immunoprecipitation, immunoblotting, immunostaining or functional studies.

### Immunofluorescence

For flow cytometry analysis of cell surface molecules, cells were detached using a non-enzymatic solution (Invitrogen), washed and stained with 10μg/ml of primary antibody. After washes in culture medium, cells were incubated with 10 μg/ml−1 FITC-labelled secondary antibody (Beckman Coulter), washed again three times and fixed with 1% formaldehyde in PBS. All incubations were performed for 30 min at 4°C. Analysis of cell-surface staining was performed using a FACScalibur flow cytometer (Becton-Dickinson, San Jose, CA, USA).

For intracellular cytometry, cells were detached using trypsin or scrapped in PBS (4°C) then fixed for 20 min at 4°C with 1% formaldehyde in PBS. After centrifugation, cells were incubated in methanol 100% for 10 min at -20°C then washed in PBS and stained with 10μg/ml of primary antibody. After washes in PBS, cells were incubated with 10μg ml FITC-labelled secondary antibody (Beckman Coulter), washed again three times and immediately analysed.

For *in-situ* labelling of p65-NFkB (Goat IgG, Santa-Cruz), cells cultured in labtek chambers were immunostained by fixing with 4% formaldehyde for 10 min followed by permeabilization with Triton X100 at 1% for 10 min. Incubation was performed in the chambers with first antibodies at 10μg/ml followed by appropriate fluorochrome coupled 2^nd^ antibodies. For CK8/18 and vimentin labelling, cells were fixed with ice-cold methanol for 10 min followed by PBS rinsing and drying for 30 min.

### Immunoprecipitation, western blot and crosslinking

Cells were lysed directly in the tissue culture flask (2 ml for a 150-cm2 flask) in lysis buffer (10 mM Tris (pH 7.4), 150 mM NaCl, 0.02% NaN3, 1 mM phenylmethylsulfonyl fluoride, 0.5 mg/ml leupeptin, 1 mg/ml pepstatin A and 10 kallikrein-inactivating units/ml aprotinin) containing 1% Brij97 (Roche Molecular Biochemicals, Meylan, France). After a 30-min incubation at 4°C, the insoluble material was removed by centrifugation at 10,000 g and the cell lysate was precleared overnight by addition of 0.005 volume of heat-inactivated goat serum and 0.025 volume of protein G-Sepharose beads (Amersham Pharmacia Biotech). Proteins were then immunoprecipitated by adding 2μg/ml of antibodies and 30 μl of protein G-Sepharose beads to 1 ml of the lysate. After a 2 hour incubation at 4°C under constant agitation, the beads were washed five times in lysis buffer. The immunoprecipitates were then separated by 5–15% SDS-polyacrylamide gel electrophoresis usually under nonreducing conditions (or after reduction of the samples when appropriate) and transferred to a PVDF membrane (Amersham Pharmacia Biotech). Western blotting on immunoprecipitates was performed using biotinylated mAbs and a Alexa Fluor 680-labelled streptavidin (Invitrogen) which was revealed with the Odyssey Infrared Imaging System (LI-COR Biosciences). Alternatively, for unlabelled primary antibodies, secondary reagents labelled with Alexa Fluor 680 or 800 nm were used. For cross-linking, the cells were incubated for 30 min at 4°C in the culture flask with 0.7mM of water soluble BS3 (Pierce, Rockford, IL) in PBS 1X. They were washed three times in PBS 1X before lysis in 1% Triton X-100 lysis buffer at 4°C and immunoprecipitation. For detection of protein tyrosine phosphorylation, the lysis buffer contained 1% Triton X-100, 50 mM tris pH 8, 150 mM NaCl, 1mM EDTA, 0.02% NaN_3_, 1mM sodium orthovanadate, 10mM NaF and proteases inhibitors. Protein phosphorylation was detected using the anti-phosphotyrosine mAb 4G10 (Upstate Biotechnology).

For subcellular detection of NFkB, a subcellular protein fractionation kit was used (Pierce Scientific, Rockford, USA).

### RNA silencing

Cells (1-3×10^5^ cells in DMEM medium) were reverse transfected with synthetic si-RNA oligonucleotides using Interferin (Ozyme) according to manufacturer's protocol.

6, 12 or 24 well cell culture plates were coated with rat tail collagen I. Plates were rinsed in PBS. To allow formation of the transfection complexes a mix of 3-5 μl of interferin, 1 μl of siRNA (10 μM) and 96 μl of free DMEM was added to each well and incubated at room temperature during 20 min to allow formation of transfection complexes. After detachment by trypsin-EDTA, cells were layered at appropriate concentration in 400μl DMEM medium with serum (the final concentration of siRNA was 20 nM).

Sequences of siRNA were either obtained from the literature or chosen according to reported criteria. Only sequences allowing inhibition of at least 75% protein expression in our system were retained for the study and are listed below. The following siRNA were synthetized by Eurogentec unless specified.

The si-RNA labelled with an asterisk were used throughout the study, the other si-RNA served as controls for specificity of the biological effects.

*si-CD53 GGAAAACAAGUGUCUGCUUdTdT

*si-Co029 GGUAUCCUAGGAGCUGUUUdTdT (see [4])

*si-EGFR CUCUGGAGGAAAAGAAAGUdTdT

si-VLA2-952D ACGCCCUUGAUACUAAAAAdTdT (see [4])

*si-VLA2-994D UCGCUAGUAUUCCAACAGAdTdT (see [4])

*si-VLA1-280D AGUUGGAUCUACCAGUUAAdTdT (see [4])

si-VLA1-171D GAAGGAAAAUGGGUGCUUAdTdT (see [4])

si-VLA1-1286D UGAACCGCUUGCUUCUUAUdTdT (see [4])

*si-Vav2.2 AGUCCGGUCCAUAGUCAACdTdT [44]

Si-Vav2.3 CAACAAGGACGUCAAGAAdTdT [44]

*si-Rac1.1 AGACGGAGCUGUAGGUAAAdTdT

si-Rac1.2 UAAGGAGAUUGGUGCUGUdTdT

*si-MyD88a AAGGAAUGUGACUUCCAGACCdTdT (Communicated by Katy Le Corf, Lyon)

si-MyD88b GGAAUGUGACUUCCAGACCUUdTdT [45]

si-TLR2b GACUUAUCCUAUAAUUACdTdT [46]

si-CD44a UAUUCCACGUGGAGAAAAAdTdT [47]

si-CD44m SMARTpool ON-TARGETplus siRNA (Dharmacon)

si-Akt-a UGCCCUUCUACAACCAGGAdTdT (targeting Akt1 and Akt2) [48] (see [4] for efficiency)

Stealth si-RNA for Co-029 and TLR2 were also purchased from Invitrogen (Carlsbad, California)

si-Co029s789 CCUGAAUUAUGUGCCUGUCUAGAUA (see [4])

*si-TLR2s UGAAGCAUCAAUCUCAAGUUCCUCA [49]

For motility measures, RNAi treatments were performed 48 hours before videomicroscopy. Experiments were repeated from 2 to 10 times to verify the robustness of the results and the stability of the measurements.

### QRT-PCR

Total RNA was purified using TRIzol reagent (Invitrogen). First, the purified RNAs were treated with DNase (Qiagen) in order to remove possible contamination with genomic DNA. Digestion was realized at 37°C for 15min in presence of 5mM MgCl2 and then the enzyme was inactivated at 75°C for 10 min. RT was performed in triplicate using the Taqman Reverse Transcription Reagents kit purchased from Applied Biosystems: 1μg of total RNA was added in a final volume of 45μl containing 1x RT buffer, 1mM dNTPs, 0.44Unit/μl of RNase inhibitor, 1μM random hexamers and 1.25 unit/μl of MultiScrib reverse transcriptase. After incubation at 37°C for 60min, the samples were heated for 5 min at 95°C to end the reaction and stored at -20°C till PCR use. cDNA (2μl) was subjected to real-time quantitative PCR using the MXPro system with Brilliant II SYBR Green QPCR MasterMix (Agilent Technologies). PCR reactions were performed for each sample and the average threshold cycle number was determined using the Mx3005P v4.10 software. Levels of specific mRNA and expressions normalized to RPL38 levels were determined using the formula 2^(Rt-Et)^ where Rt is the threshold cycle for the reference gene (RPL38), and Et is the threshold cycle for the experimental gene (ΔΔCT method). Data are thus expressed as arbitrary units. Sequences of primers used: Snail (F: 5'-CCTCCCTGTCAGATGAGGAC-3’, R: 5'-CC AGGCTGAGGTATTCCTIG-3’), Twist (F: 5'-GGA GTCCGCAGTCTTACGAG-3’, R: 5'-TCTGGAGGACC TGGTAGAGG-3’), Zeb2 (F: 5'-TTCCTGGGCTACG ACCATAC-3’, R: 5'-TGTGCTCCATCAAGCAATTC-3’), Slug (F: 5'-GGGGAGAAGCCTTTTTCTTG-3’, R: 5'-TCCTCATGTTTGTGCAGGAG-3’).

### Videomicroscopy

Analysis of 2D cell motility on collagen I films was performed using phase contrast on an inverted microscope (Axiovert 200; Zeiss, Oberkochen, Germany) equipped with an environmental chamber with 5% CO2 at 37°C. Cells were seeded in 24 well plates (5000 cells/well) in DMEM supplemented with 10% FCS, glutamax and antibiotics. The microscope was driven by the Metamorph software (Roper Scientific) and images were recorded with a Coolsnap HQ camera (Roper Scientific). Stacks of phase contrast images were collected every 15 min for 24 h at x200 magnification. Cell migration was quantified using the manual tracking plugin of ImageJ [50]. Raw data were transferred to Excel for speed calculations and statistical analysis was performed by the non-parametric Mann-Whitney test in Graphpad. For each position at least 10 cells were analyzed. In addition to various siRNA, Cetuximab (25 μg/ml), the EGFR inhibitor AG1478 (Merck, Germany) at 5μM, PD98059 for Mek1 (20μM), U0126 (Promega) at 10μM for the MAPK Erk, SB203580 (Calbiochem) at 20 nM for the MAPK p38, D-JNKI-1 targeting JNK1 (a gift of Xigen SA) at 3μM were used in motility experiments as well as the TLR2 ligand B. subtilis peptidoglycan, PGN (InvivoGen, San Diego).

### *In vivo* experiments

Balb/c nude mice were injected with 5.10^6^ SW480 tumor cells subcutaneously in the back on day 0. Mice were divided in two groups which were injected either with mycoplasma infected or mycoplasma free cells, each group comprising 5 mice. Experiments were conducted according to the French veterinary guidelines and those formulated by the European Commission for experimental animal use (L358-86/609EEC) and were approved by Inserm (National Institute for health and medical research, France).

### Mass spectrometry protein identifications

Immunoisolation of Co-029-containing complexes and In-gel Tryptic Digestion—For identification of Co-029-associated molecules, cells were lysed *in situ* (150cm^2^ flasks) with 3 lysis buffer/flask containing 10 mM Tris, pH 7.4, 150 mM NaCl, 0,02% NaN3, 1% Brij97 in the presence of protease inhibitors. Insoluble material was removed by centrifugation at 12,000 g for 15 min, and the lysates were precleared three times successively with NHS-activated Sepharose™ High Performance, *GE Healthcare* beads coupled to BSA and to goat serum (Sigma). Isolation of Co-029 or CD9 containing complexes was performed using beads coupled to mAb TS29.2 or Ts9 respectively. The beads were washed five times with lysis buffer, and the proteins were eluted by boiling for 3 min in in Laemmli buffer containing 0.1% SDS. Brij97 was replaced by 1% Triton X100 for crosslinked extracts. The proteins were separated by 5–15% SDS-polyacrylamide gel electrophoresis under non-reducing conditions. For mass spectrometry analysis, the gels were stained with colloidal Coomassie Blue (Bio- Rad). The proteins were excised and destained in 200 μl 0, 1M NH4HCO3/acetonitrile v/v for 20 min, centrifuged and swollen in H2O repeatedly until complete destaining. Gel pieces were then incubated in 150 μl 100% acetonitrile for 10 min and dried. This was followed by rehydratation in 100 mM ammonium bicarbonate containing 10 mM DTT for 45 min at 56°C. After cooling to room temperature, the DTT solution was replaced with 55 mM iodoacetamide in 100 mM ammonium bicarbonate for 30 min at room temperature in the dark.

The gel pieces were washed in 300 μl 0, 1M NH4HCO3/acetonitrile v/v for 15 min, dehydrated in 100% acetonitrile, and dried. For trypsin digestion, the Trypsin Profile IGD Kit-PP0100 (*Sigma*) for in gel digest was used according to manufacturer's instructions. Following enzymatic digestion overnight at 37°C, and supernatant retrieval, the gels fragments were extracted twice by addition of 20 μl of an Acetonitrile/5% formic acid 70/30 v/v and incubation for 20 mn at 37°C and supernatants were pooled, dried and rehydrated in Acetonitrile/formic acid/H2O 3/0.5/96 v/v.

LC-MS/MS analyses were performed using an ESI linear ion trap-Orbitrap hybrid mass spectrometer (LTQ-Orbitrap Velos, *Thermo Fisher Scientific, Bremen, Germany*) coupled on line with a nano-HPLC system (Ultimate 3000; *Dionex*) for liquid chromatography. 5 μl digested protein were Injected in the system by using a pre-concentration column (C18 trap column - PepMap C18, 300 μmID×5 mm, 5μm particle size and 100 Å pore size; *Dionex*). The nano-column used in this study was a PepMap C18 reverse phase (Acclaim pepmap RSLC 75 μm x 15 cm, nanoViper C18, 2 μm, 100 Å). A linear 45 min gradient (flow rate, 300 nl/min) from 4 to 55% acetonitrile in 0.1% (v/v) was applied. After the acquisition of a full MS scan by the Orbitrap at high resolution (30000 resolution, *m*/*z* range were 380–1700 Da) in the first scan event, the five most intense ions present were subsequently isolated for fragmentation (MS/MS scan). The collision energy for the MS/MS scan events was pre-set at a value of 35%, isolation window was set at 3 Da, Dynamic exclusion option was enabled. The capillary voltage was set at 1.6kV and the temperature was 275°C.

The data were analyzed by the Proteome Discoverer 1.4 software. The database is human (Swiss-Prot), the mass error for the precursor ions (full MS) is less than 10 ppm (error_ppm_ = (m/z_experimental_ - m/z_exact_) x 10^6^/ m/z_exact_. Mass error for ions from the MS/MS spectra is reported less than 0.6 Da. Peptides mass is searched between 350 Da and 5000 Da with time retention from 10 min to 50 min. A miss cleavage site is tolerated. Dynamic modification was enabling for N_ter_ acetylation, oxidation of methionine and histidine, carbamidomethylation for amino acids, aspartic acid and glutamic acid. Dynamic modification BS^3^ was added for lysine in covalent bond experiments. Static carbamidomethyl modification of cysteine was enabled. Peptide identifications were validated by determination of false positives by Target decoy PSM validator. It is high if the false positive rate (FDR or false Discovery rate) is less than 1%, low if the FDR is greater than 5% and average (medium between 1 and 5 %). Peptide identification Xcorr were calculated by the correlation of MS/MS experimental spectrum compared with the theoretical MS/MS spectrum generated by the Proteome Discoverer 1.4 software. For analysis of mycoplasma proteins, a compilation of 91 Uniprot databases was used.

A relative quantitation was performed with the Proteome Discoverer integrated label free method which consists in comparing the mean peaks area of the three best peptides to a given protein from one sample to another. The method of calculation is three dimensional relying on retention time, ion intensity and m/z ratio of the peptide, with a mass error lower than 2 ppm.

## SUPPLEMENTARY MATERIALS FIGURES AND TABLES







## Abbreviations

ERM: Ezrin-Radixin-Moesin
RNAi: RNA interference
RTK: Receptor Tyrosine Kinase
siRNA: small interfering RNA
TLR: Toll Like Receptor.
