# Supplementary material for: Multi-factorial modulation of colorectal carcinoma cells motility – partial coordination by the tetraspanin Co-029/tspan8
Source: Oncotarget. 2017 Mar 16;8(16):27454–70. doi: 10.18632/oncotarget.16247 (PMC5432348; doi:10.18632/oncotarget.16247)
Supplement: Supplementary file 1 [file oncotarget-08-27454-s001.pdf]

Multi-factorial modulation of colorectal carcinoma cells motility – partial coordination by the tetraspanin Co-029/tspan8

SUPPLEMENTARY MATERIALS

SUPPLEMENTARY FIGURES, MOVIES AND TABLES

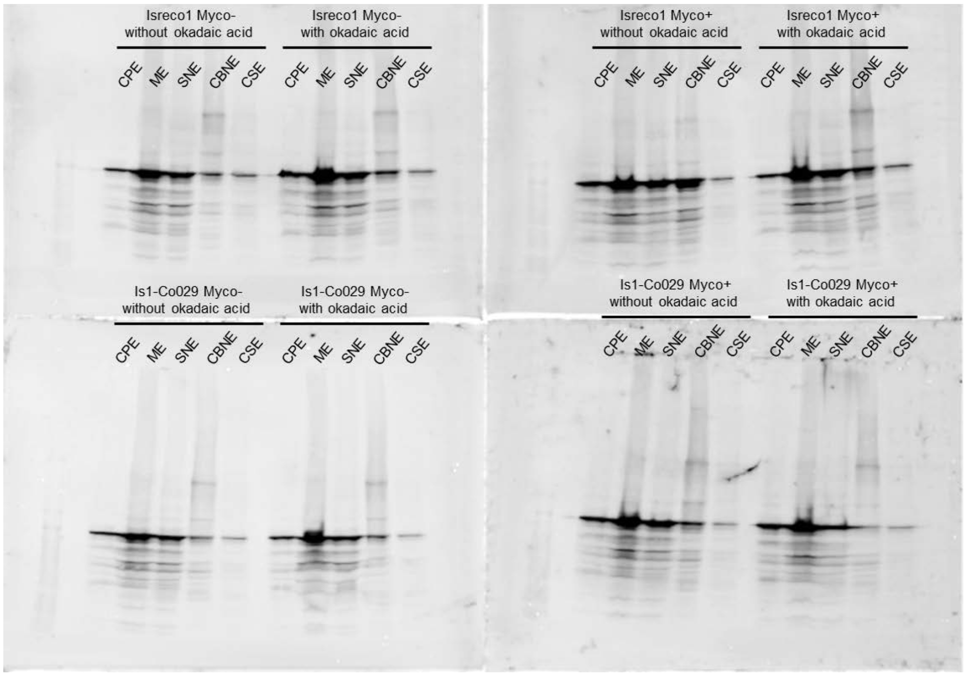

Supplementary Figure 1: Subcellular localization of p65NFkB. Western blot with an anti-p65NFkB mouse monoclonal antibody revealed by a goat anti-mouse (Alexa Fluor 800 nm) of subcellular protein extracts from Isreco1 and Is1-Co029 cells, either mycoplasma infected (Myco+) or free (Myco-) and incubated or not with okadaic acid (10 nM). Cytoplasmic (CPE), membrane (ME), soluble nuclear (SNE), chromatin-bound (CBNE) and cytoskeletal (CSE) protein extracts were tested in the different conditions. All cells were cultured on a collagen I substrate.

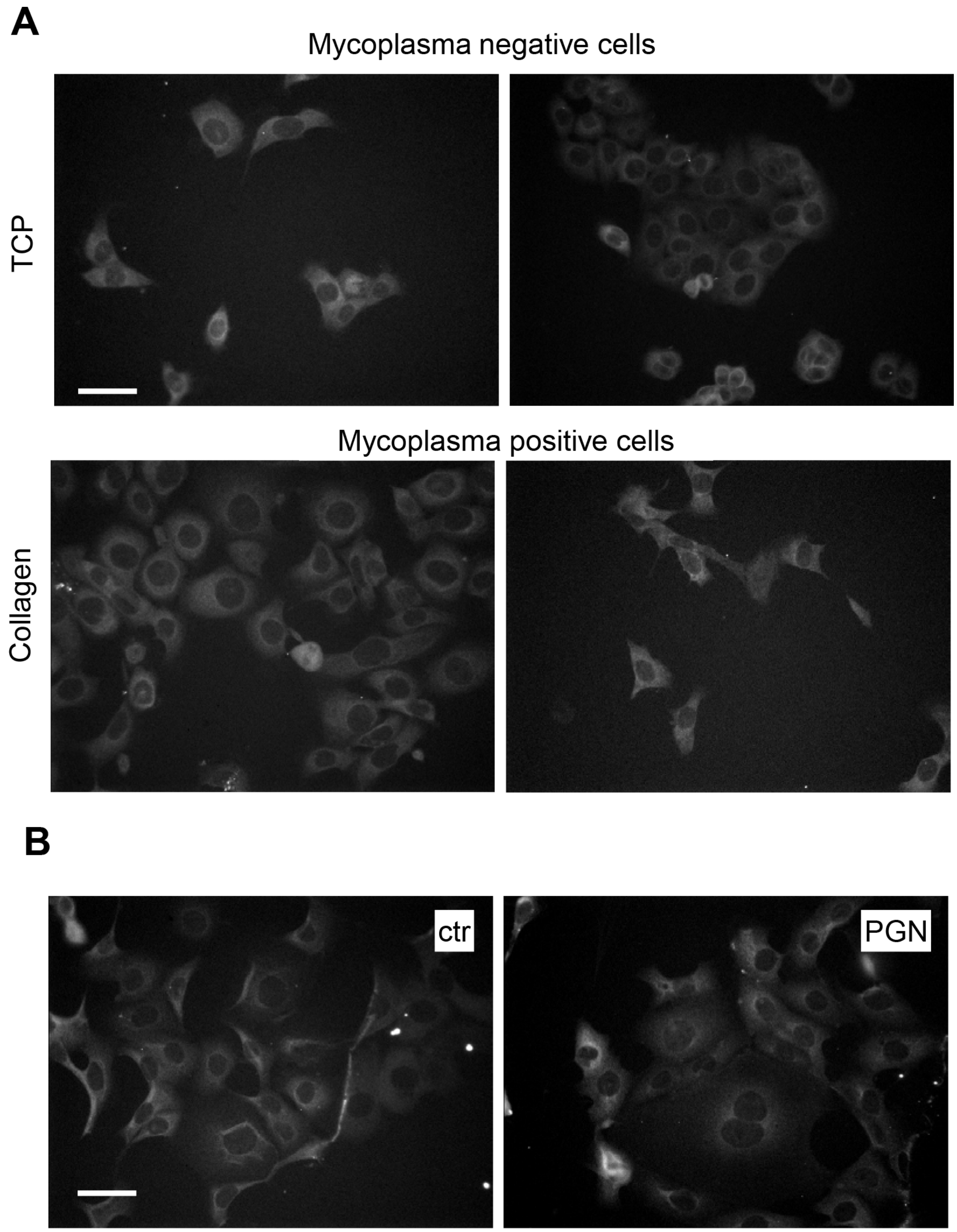

Supplementary Figure 2: p65NFkB cellular localization in Is1-Co029 cells. (A) p65NFkB remains cytoplasmic independently of mycoplasma status or addition of a collagen I. **(B)** The TLR2 ligand PGN does not induce the translocation of p65NFkB to the nucleus. Scale bar 50µm.

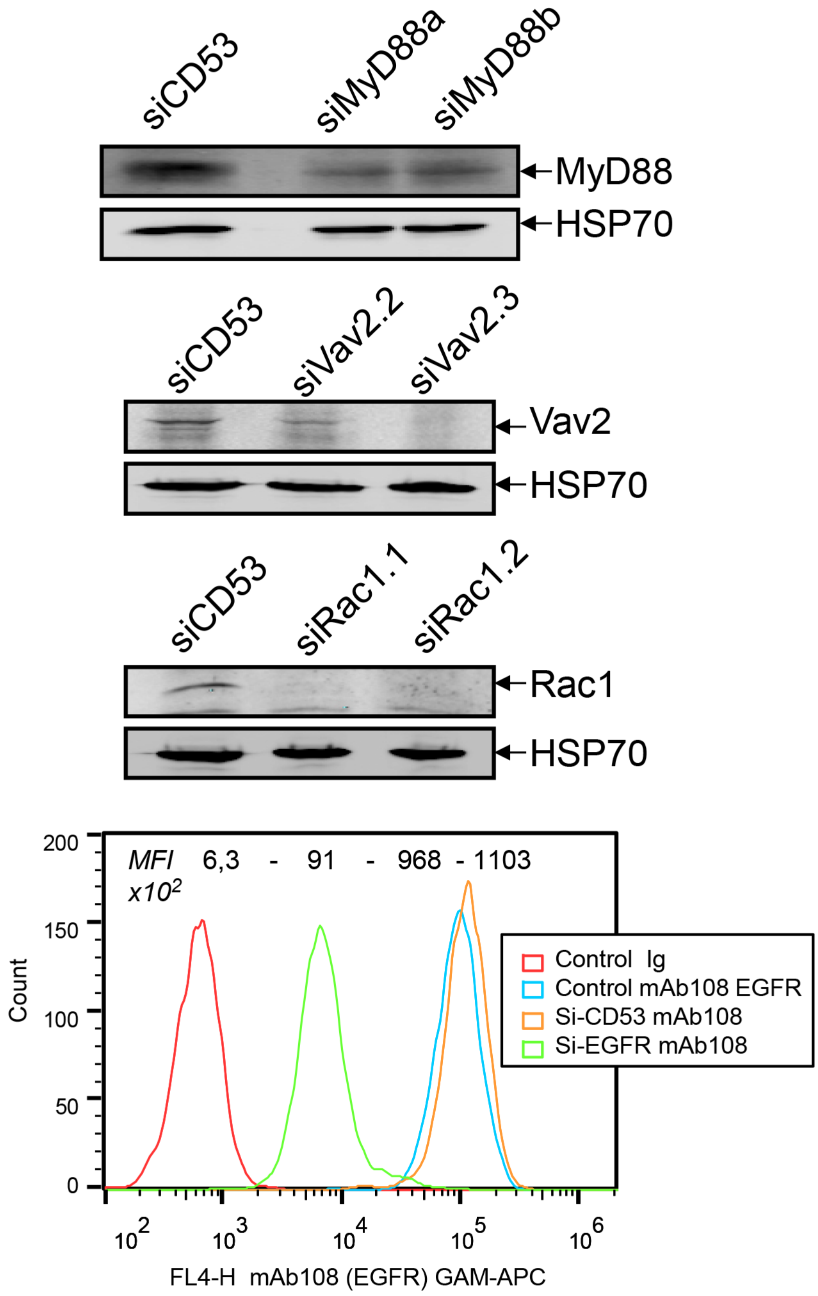

Supplementary Figure 3: Efficiency on protein expression of siRNA to MyD88, Vav2, Rac1 (western blot) and to EGFR (flow cytometry).

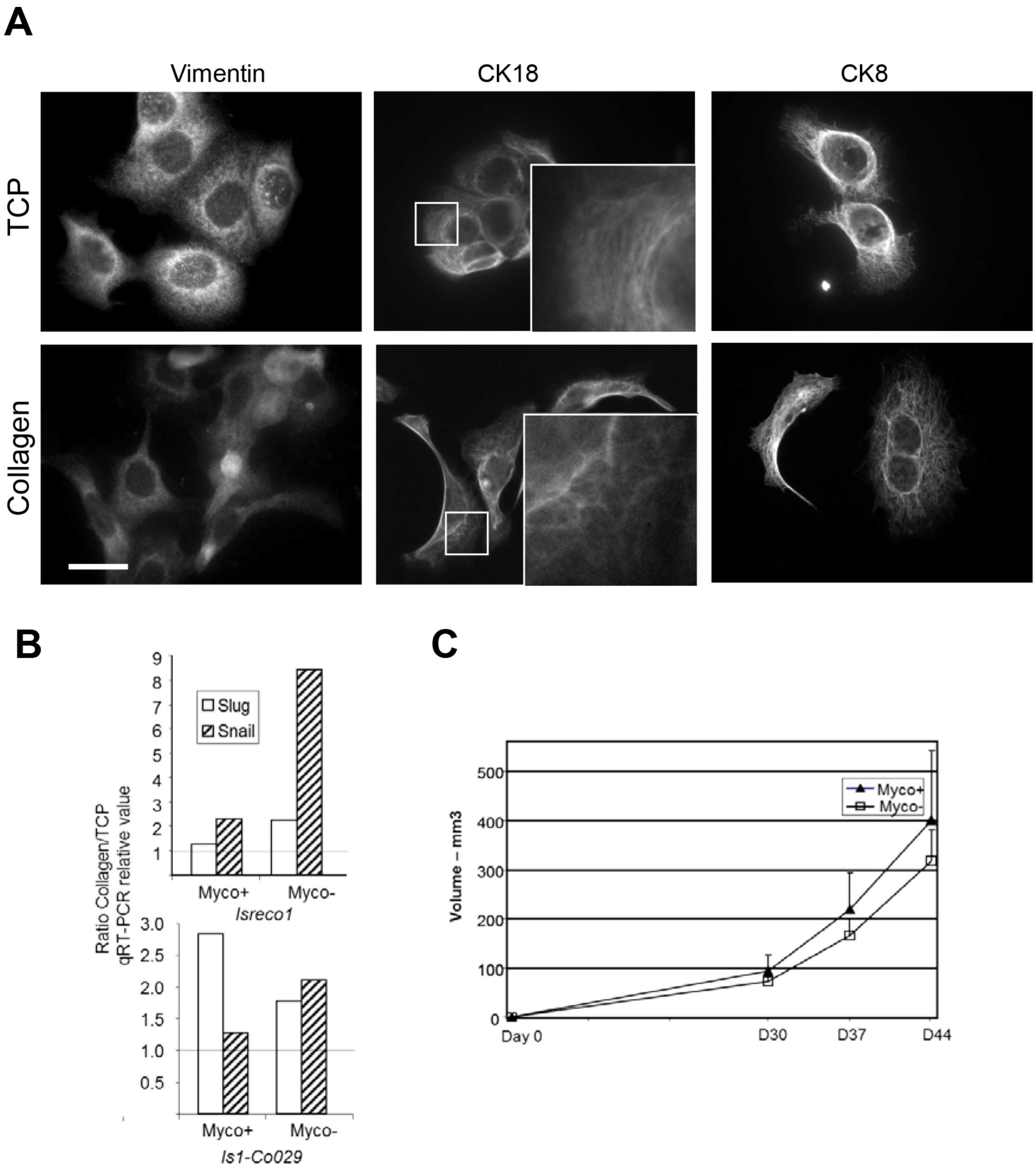

Supplementary Figure 4: EMT markers and *in vivo* tumor growth in relation to mycoplasma infection. **(A)** Effect of collagen on intermediate filaments expression of Isreco 1 cells (mycoplasma infected). Despite differences in cell morphology of cells cultured on collagen I compared to TCP (tissue culture plastic), vimentin labeling remains diffuse and punctuate whereas cytokeratins 8 and 18 are still strongly expressed with a fibrillar aspect (magnified in the insert of CK18 pictures). Scale bar 25µm. **(B)** Slug and Snail mRNA expression in Isreco cells. Mycoplasma infected (Myco+) or free (Myco-) cells were cultured with or without substrate. Results are shown as ratio of collagen vs TCP relative amounts. Culture on collagen induces a variable increase of Slug and Snail mRNA. **(C)** The *in vivo* growth of tumors is not significantly modified by the presence (Myco+) or absence (Myco-) of mycoplasma in subcutaneously injected SW480 cells.

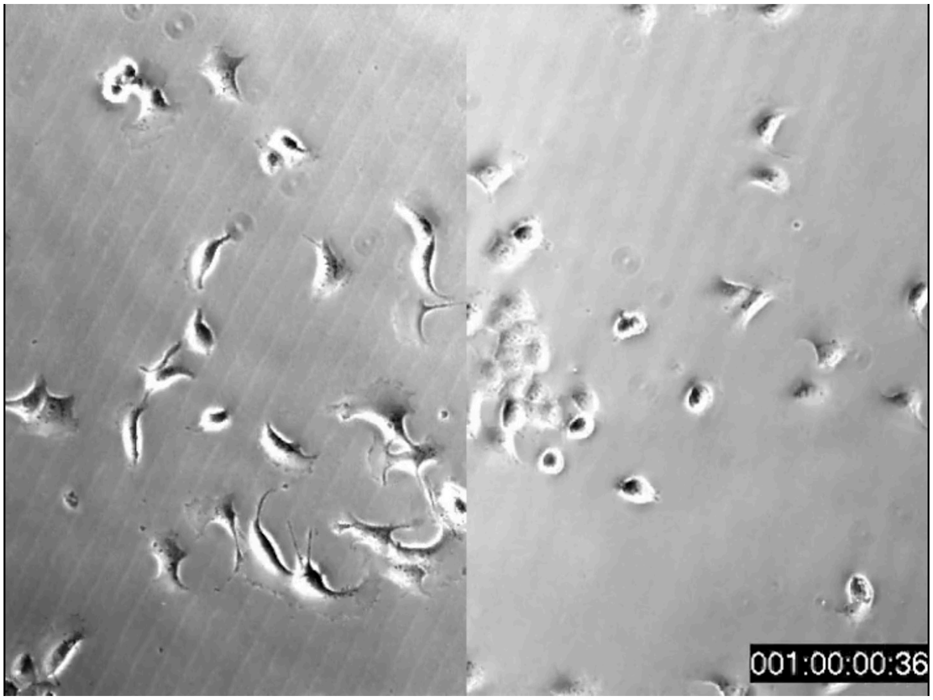

Supplementary Movie 1: This file contains Supplementary Movie 1 showing side by side comparison of cell motility on collagen I of Isreco1 cells infected by mycoplasma (left) and cells treated with BM-Cyclin (checked for the absence of mycoplasma) (right). Mycoplasma infected cells move rapidly with an elongated arc or fan shape whereas mycoplasma free cells move only slightly and as a consequence remain together upon division.

See Supplementary Movie 1

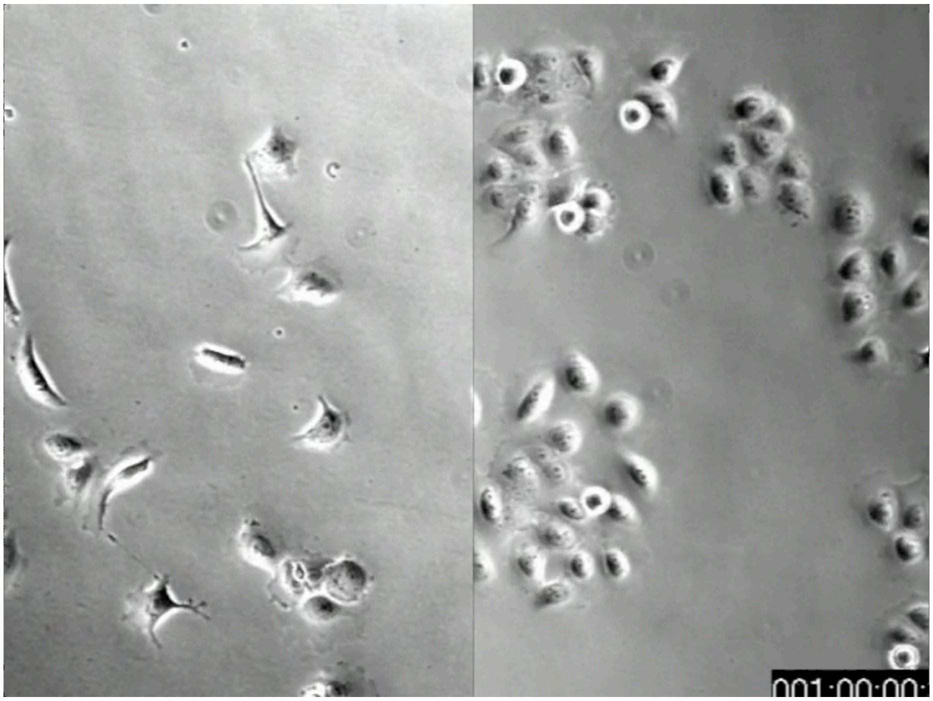

Supplementary Movie 2: This file contains Supplementary Movie 2 showing side by side comparison of cell motility of mycoplasma free Isreco1 cells with PGN (a lipopeptide from the mycoplasma membrane) (left) and without PGN (right). PGN induces cell motility of mycoplasma free cells on collagen I.

See Supplementary Movie 2

Supplementary Table 1: Seventeen Mycoplasma hyorhinis proteins found by MS analysis as contaminants of tetraspanins complexes immunoprecipitates

Accession	Description	Mycoplasma hyorhinis strain	Coverage	#Unique Peptides	#Peptides	#PSMs	S-Area	AAs	MW [kDa]
E0TK89	Uncharacterized lipoprotein	strain HUB-1	52,79	11	38	61	1,66E+08	771	88
K7X9H3	High affinity transport system protein p37	SK76	49,88	6	20	30	2,05E+08	403	46
E0TLD6	Uncharacterized protein	strain HUB-1	23,68	5	14	17	6,61E+07	722	83
K7X8P8	Cytosol aminopeptidase pepA	SK76	36,68	5	12	14	3,32E+07	458	51
E0TLG9	Uncharacterized protein	strain HUB-1	13,62	4	10	14	1,07E+08	947	109
K7X8S8	ABC transporter xylose-binding lipoprotein	SK76	38,41	4	14	21	5,35E+07	453	50
K7XZ32	Enolase	SK76	29,11	4	8	9	2,63E+07	450	49
E0TK90	Uncharactized lipoprotein	strain HUB-1	28,95	3	20	23	4,52E+07	753	85
E0TKQ5	46kDa surface antigen	strain HUB-1	27,07	3	11	16	6,53E+07	447	49
E0TL59	101 kDa protein	strain HUB-1	8,72	3	8	8	4,10E+07	1044	119
K7X3C4	Uncharacterized protein	SK76	26,96	3	5	5	2,92E+07	230	26
E0TKF0	Phosphoglycerate kinase	strain HUB-1	49,25	2	13	14	6,78E+07	402	44
E0TL30	ATP synthase subunit b	strain HUB-1	26,46	2	5	5	8,13E+06	189	22
E0TLN9	Uncharacterized protein	strain HUB-1	19,86	2	15	18	6,67E+07	851	96
K7X1Y7	Uncharacterized protein	SK76	13,03	2	3	4	1,64E+07	284	32
K7X3I4	Uncharacterized protein	SK76	19,86	2	15	15	8,94E+07	851	96
K7X9I5	Uncharacterized protein	SK76	15,86	2	12	12	1,32E+07	946	108

Supplementary Table 2: Integrins chains found by mass spectrometry analysis in the tetraspanin complexes of Isreco1 and Is1-Co029 cells cultured either on tissue culture plastic or on collagen I after CD9 (Ts9 mAb) or Co-029/Tspan8 (Ts29.2 mAb) immunoprecipitation (IP)

Substrate	Tissue Culture Plastic									Collagen I								
	Isreco1 IP CD9			Is1-Co029 IP CD9			Is1-Co029 IP Co029			Isreco1 IP CD9			Is1-Co029 IP CD9			Is1-Co029 IP Co0299		
	Peptides		S-Area	Peptides		S-Area	Peptides		S-Area	Peptides		S-Area	Peptides		S-Area			
Integrins Chains	*Total*	*Unique*		*Total*	*Unique*		*Total*	*Unique*		*Total*	*Unique*		*Total*	*Unique*		*Total*	*Unique*	
ITGA2				2	1	8,88E+05	6	1	9,13E+06	3	1	4,44E+06	2	1	2,39E+06			
ITGA3	4	2	6,37E+08	5	1	1,56E+09	31	18	1,04E+09	23	14	1,56E+09	23	13	1,89E+09	19	10	4,18E+08
ITGA6	6	1	2,40E+08	12	3	3,33E+08	28	14	1,69E+08	33	15	4,77E+08	41	21	3,50E+08	29	12	7,62E+07
ITGAV	1	1	2,70E+06	12	6	2,63E+07	17	5	1,30E+07				8	3	1,03E+07	14	4	1,07E+07
ITGB1	15	7	2,77E+09	33	18	3,07E+09	33	19	1,72E+09	29	18	2,72E+09	28	17	3,03E+09	27	16	2,43E+09
ITGB4	9	2	1,65E+08	4	1	3,27E+08	19	9	9,86E+07	39	21	4,28E+08	30	19	4,19E+08	35	18	1,25E+08
ITGB5				2	1	3,95E+06	7	2	1,82E+07							8	1	7,96E+06
ITGB8																4	1	3,90E+06
